# Society Reports—Atlanta Medico-Chirurgical Association

**Published:** 1878-05-20

**Authors:** 


### SOCIETY REPORTS.

### ATLANTA MEDICO-CHIRURGICAL ASSOCIATION.

Reported by Dr. Olmstead.

Monday evening, April 1st, Dr. Roach in the chair.

Dr. Knott exhibited a specimen of a female foetus which he removed from a negro woman who had been in labor over 100 hours. The patient was a mulatto, aged 35 years, and had borne two children previously, experiencing some difficulty with one of them.

When he arrived, found the woman almost moribund, pulse extremely feeble and labor pains entirely subsided.

On examination found the occiput presenting to the symphysis. The head very large, and the bones of the craneum so completely ossified that no fontanelles existed. An effort was made to introduce the forceps, but was found impracticable.

In consultation with Dr. A. R. Alley, the operation of craniotomy was decided upon. The operation proved to be exceedingly difficult by reason of the entire ossification and density of the cranium. After long and laborious efforts, and before the operation was complete, the woman began to sink, and though stimulants were administered, and every effort made to sustain her, she expired undelivered.

Being anxious to see the child, and ascertain fully the nature of the difficulty, he procured the consent of the friends to remove the fœtus by cæsarian section, which he did, and extracted a fœtus of extraordinary size, as seen in the specimen presented. Length, 22½ inches; the diameters of the scalp relatively disproportioned to those of the pelvis, and the weight of the child at least eighteen or twenty pounds!

It was evidently a mechanical impossibility for the child to have been born. Nor is it likely that it could have been delivered by the perforator or by evisceration on account of the extraordinary size of the fœtus. Dr. Knott expressed the opinion that the cæsarian section in such cases would be not only justifiable, but would be clearly indicated as affording the best chance for both mother and child. It was in the highest degree probabable that, with the state of innertia in which he found the womb, if he had succeeded in delivering with the forceps or the crotchet, the woman would have died from uterine hemorrhage; whereas, the cæsarian section would have furnished a better chance to control the hemorrhage by manipulation.

Dr. Alley, who assisted Dr. Knott in this case, confirmed the facts given by him, and concurred in the views expressed. He regarded the case as one of prolonged gestation.

Dr. H. B. Lee agreed with Dr. Knott's conclusions as to what could be done at the *time* and under the circumstances that he saw the case. The excessive distension interfering with locomotion, rest and nutrition, continuing probably for several weeks in this case, had suggested to his mind the propriety of the induction of premature labor in similar cases.

Dr. E. J. Roach remarked that the question as to the propriety of performing so grave an operation as the cæsarian section in a woman who had previously given birth to children, is one difficult to decide. He would certainly not do so without first exhausting all other instrumental methods.

Dr. Knott stated that though the cæsarian section was a very grave operation, and to be resorted to only in very exceptional cases, it was too frequently postponed until too late.

Dr. A. J. Pinson presented to the Association a specimen of worms (ascaris lumbrocoides), 73 in number, discharged in one day. Worm fever was well marked in this case. Treatment—

℞ Saccharated calomel.

Santonin, aa grs ij.

Ft four powders.

Take one every four hours.

Dr. Pinson also reported a case of measles wherein the eruption was visible on tho tongue and fauces five days before it appeared on the skin.

—

*Monday, April* 22.

Dr. Grawford in the chair.

Dr. Asher reported a case of strangulated inguinal hernia, occuring in a young man aged seventeen, who had been the subject of congenital hernia. Recently, in carrying a heavy weight, the hernia suddenly increased in size—a large tumor appearing in the scrotum. The pain and other symptoms becoming worse, the Doctor was called in a few days after the apperance of the tumor. He found great difficulty in making a diagnosis as to what the contents of the tumor were. There was a large, soft mass, but no distinctive evidence of the presence of the intestine could be obtained. Dr. Willis Westmoreland saw the case with him on Friday, both agreed as to the difficulty of making a diagnosis as to what the contents of the tumor were.

On Sunday it was decided that an operation should be performed, which was done by Dr. Westmoreland, who cut down upon the tumor, which was found to consist of a large mass of the omentum, with only a small portion of intestine (about one third of an inch), had descended.

The stricture was divided, and the gangrenous portions of the omentum having been removed, the remaining parts were returned to the abdominal cavity. The patient died haJ an hour after the completion of the operation, having never rallied, even from the effects of the ether which was used. He had been in very good condition at the commencement of the operation, in regard to his pulse condition of the skin, etc.

Dr. Asher concluded his report by remarking that, to his mind, the important point in this case was the difficulty in making a diagnosis; and he would like to know how he could have settled in advance the question as to what this large, soft mass in the scrotum was. An interesting discussion followed as to what was the cause of death in this case, there having been no symptoms of peritonitis before the operation was undertaken, and as the death took place half an hour afterwards, this complication could not have been in force, as a cause. Dr. Asher was satisfied it was not due to the anaesthetic used; he thought the patient died from the "shock of the operation." In reply to a question as to the post mortem appearance of the body, he stated that only a small slough, about the size of a pea, indicated a leison of the bowels.

Dr. J. J. Knott reported a case presenting the following symptoms: A gentleman had fever for five days, complained of headache, soreness in the muscles of the back, etc. At the expiration of that time, an eruption appeared, papular in character, and there was a cessation of the fever. The eruption, at first papular, became umbilicated, patient complaining of severe itching. Dr. Alley stated that he had seen this case with Dr. Knott, and he considered it a well marked case of variola.

